# A136 A RARE CAUSE OF AUTOIMMUNE ATROPHIC PANGASTRITIS COMPLICATED WITH GASTRIC OUTLET OBSTRUCTION

**DOI:** 10.1093/jcag/gwab049.135

**Published:** 2022-02-21

**Authors:** D Akhtar, M A Donaldson, N H Akhtar, H Yang, F Donnellan

**Affiliations:** 1 Medicine, The University of British Columbia Faculty of Medicine, Vancouver, BC, Canada; 2 Vancouver General Hospital, Vancouver, BC, Canada; 3 The University of British Columbia Department of Medicine, Vancouver, BC, Canada; 4 School of Medicine, University College Dublin, Dublin, Ireland

## Abstract

**Background:**

Chronic gastritis comes in two well recognized forms: environmental, which is most commonly antral or multifocal in distribution and is typically caused by Helicobacter Pylori (HP) infection, and autoimmune gastritis(AIG), which affects the corpus and fundus. Presented here is a case of autoimmune atrophic pangastritis(AIAP).

**Aims:**

To increase awareness of a rare condition with limited data on available treatment modalities.

**Methods:**

Case Report

**Results:**

A 68-year old female with autoimmune hypothyroidism, presented with weight loss and elevated anti-TTG serology. Index esophagogastroduodenoscopy (EGD) biopsies demonstrated chronic non-specific gastritis limited to the antrum. Strict gluten free diet adherence was initiated. Testing for HLA revealed HLADq2 and HLADq8 negativity but HLADq2.5 positivity. Subsequent EGD showed a markedly atrophic appearing gastric body. The corresponding biopsies demonstrated persistent moderately chronic active gastritis with severe atrophy now involving the body, fundus and antrum. The biopsies were negative for HP. Notably, there was a lack of ECL-cell hyperplasia and the number of antral G cells appeared decreased. Anti-parietal cell antibody serology was positive with a titre of 1:80. Despite combination therapy with budesonide and mesalamine and treatment for HP given persistent symptoms, the patient’s course was further complicated by gastric-outlet obstruction (GOO). Urgent EGD biopsies showed pyloric stenosis requiring dilation. Endoscopic Ultrasound (EUS) guided biopsies were negative for malignancy. The patient was started on corticosteroids and azathioprine(AZA). Most recently, an EGD on AZA, continued to demonstrate severe chronic active pangastritis now with intestinal metaplasia involving the body. Corticosteroid therapy was reinitiated with a plan to start mycophenolate mofetil (MMF).

**Conclusions:**

AIAP is a rarely described entity, not well documented in the literature. An eight patient case series reported a distinctive form of atrophic gastritis that was independent of HP infection with the absence of neuroendocrine hyperplasia that involved the body and antrum. Thyroid disease, specifically Hashimoto thyroiditis is present in about 40% of patients with AIG. Additionally, AIG progression to atrophic gastritis with intestinal metaplasia confers an increased risk for gastric adenocarcinoma in more than 10% of patients.

Limited literature exists regarding the management of AIAP. Pediatric data suggests the use of prednisone and/or azathioprine for AIAP. Furthermore, a recent case report of AIAP demonstrated clinical and endoscopic remission with MMF. Currently, no guidelines exist for the treatment, screening and monitoring of patients with AIAP. This case report presents a rare case of AIAP refractory to AZA complicated with GOO and adds to the little literature that exists.

**Funding Agencies:**

None

